# Lemon essential oil ameliorates age-associated cognitive dysfunction via modulating hippocampal synaptic density and inhibiting acetylcholinesterase

**DOI:** 10.18632/aging.103179

**Published:** 2020-05-11

**Authors:** Bonan Liu, Jiayuan Kou, Fuyan Li, Da Huo, Jiaran Xu, Xiaoxi Zhou, Dehao Meng, Murtaza Ghulam, Bobkov Artyom, Xu Gao, Ning Ma, Dong Han

**Affiliations:** 1 Department of Biochemistry and Molecular Biology, Harbin Medical University, Harbin 150081, China; 2 Basic Medical Institute of Heilongjiang Medical Science Academy, Harbin 150081, China; 3 Translational Medicine Center of Northern China, Harbin 150081, China; 4 Heilongjiang Provincial key Laboratory of Genetically Modified Model Animal, Harbin Medical University, Ministry of Education, Harbin 150081, China; 5 China Key Laboratory of Preservation of Human Genetic Resources and Disease Control in China (Harbin Medical University), Ministry of Education, Harbin 150081, China

**Keywords:** lemon essential oil, aromatherapy, cognitive dysfunction, Alzheimer’s disease, synaptic plasticity

## Abstract

The lemon essential oil (LEO), extracted from the fruit of lemon, has been used to treat multiple pathological diseases, such as diabetes, inflammation, cardiovascular diseases, depression and hepatobiliary dysfunction. The study was designed to study the effects of LEO on cognitive dysfunction induced by Alzheimer’s disease (AD). We used APP/PS1 double transgene (APP/PS1) AD mice in the experiment; these mice exhibit significant deficits in synaptic density and hippocampal-dependent spatial related memory. The effects of LEO on learning and memory were examined using the Morris Water Maze (MWM) test, Novel object recognition test, and correlative indicators, including a neurotransmitter (acetylcholinesterase, AChE), a nerve growth factor (brain-derived neurotrophic factor, BDNF), a postsynaptic marker (PSD95), and presynaptic markers (synapsin-1, and synaptophysin), in APP/PS1 mice. Histopathology was performed to estimate the effects of LEO on AD mice. A significantly lowered brain AChE depression in APP/PS1 and wild-type C57BL/6L (WT) mice. PSD95/ Synaptophysin, the index of synaptic density, was noticeably improved in histopathologic changes. Hence, it can be summarized that memory-enhancing activity might be associated with a reduction in the AChE levels and is elevated by BDNF, PSD95, and synaptophysin through enhancing synaptic plasticity.

## INTRODUCTION

Neurodegenerative diseases of the nervous system are always related to impaired learning and memory ability, eventually inducing to cognitive disorders [1]. AD is the leading cause of cognitive deficits and the most common neurodegenerative disease of aging [2]. It is pathologically characterized by a gradual impairment of learning and memory, accompanied by pathological characteristics such as tau protein neurofibrillary tangles and accumulation of amyloid plaques [3, 4]. Interaction of Aβ activates local oxidative and inflammatory responses [5]. Cognitive impairment of AD is correlated with a synaptic impairment in the cholinergic system [6], because of an insufficiency of ACh due to an increased content of AChE in the brain. Additionally, synaptic loss and impairment are the most foremost hallmarks of cognitive deficits in AD [7]. The symptoms of AD can be elucidated by a loss of plasticity [8], which may have negative dendritic ramifications in processes such as synaptic remodeling, long-term potentiation (LTP), neurite extension, axonal sprouting, neurogenesis and synaptogenesis. To date, few studies have revealed that medical compounds could treat working memory impairment and decremental LTP and have discussed the potential mechanisms [9].

Aromatherapy and essential oils have been proposed as a underlying preventive and treatment strategies for anti-aging and neurodegenerative disorders such as AD [10]. Aromatherapy is a safe and professional treatment using the essential oils, which are extracted and processed from a plant’s flower, bark, leaf, root or peel [11–13]. Studies have shown that Linalool, a major volatile monoterpene component of lavender essential oil, has potent neuroprotective effects in scopolamine-induced AD mice [14]. Coconut oil can protect against neuron differentiation from amyloid toxicity [15]. Additionally, researchers have reported that palm fruit bioactives could modulate the neuroinflammatory events associated with IL-1β-activated human astrocytes in vivo [16]. Declining sensory functions are also common in neurodegenerative disorders, including AD [17]. Essential oil is volatile and it may play a role in cognitive improvement through olfactory pathways [18]. However, the underlying mechanisms by which essential oil act remain obscure.

Natural products have long been used as solutions to treat the pathogenesis associated with cognitive impairment in the form of essential oils. Some natural bioactive compounds currently obtainable for the treatment of AD are acetylcholinesterase inhibitors (AChEIs) [19]. Rivastigmine and galantamine were approved to treat AD and were designed from the lead composite physostigmine, a natural AChEI alkaloid [20]. Among plant resources, essential oils contain hundreds of lipophilic and low-molecular-mass components that can pass through the blood-brain barrier (BBB) and cell membranes [21]. Some non-alkaloidal and AChEIs have been gained from natural plants, including flavonoids and terpenoids in essential oils. However, only a small number of resources has been found to improve synaptic plasticity [22] and neurogenesis despite the great efforts spent through high-throughput and high-content screening thus far [23]. Synaptic plasticity is mediated by multiple extracellular factors and intracellular pathways. Furthermore, upon stimulation of growth factors, neurotrophins, or other morphogens, proper activation of typical intracellular signaling pathways, including ERK and AKT kinase cascades, is critical [24]. Recently, several natural products extracted from herbs have been reported to enhance neurogenesis, revealing the potential mechanism for the cognition-enhancing effects of these herbs [25]. *Acori tatarinowii* has long been a medicine commonly used in traditional Chinese formulas to treat brain diseases, such as dysmnesia, stroke and dementia. Recent pharmacological investigations have revealed that *Acori tatarinowii* has neuroprotective effects [26, 27] and prompts learning and memory in aged, dysmnesia mice [28, 29]. However, whether there are other plants that play a similar role is not known.

We noted that LEO (derived from *Citrus limon*) can increase neuroprotective function, memory [30], and attention levels in patients with AD [31], cognitive test anxiety among nursing students [32], and mood. Some randomized, double-blind, placebo-controlled trials have also revealed anxiolytic-like effects in humans with the extract of lemon [33, 34]. Its health-related biological activity is not only associated with Vitamin C but also related to flavonoids with antiallergic, antioxidant, anti-inflammatory, anticancer and anticarcinogenic actions [35]. Recent pharmacological studies have also revealed that members of the genus typically express some monoterpenes that provide their “lemon” flavor and odor---most notably, linalool, limonene, α-pinene, β-myrcene, eriodictyol [35] and their derivatives, as well as some more typical monoterpenes expressed by related genera [36]. The monoterpene factors may possess multiple effects directly related to brain function, which include: cholinesterase inhibition activity by essential oils [37]. The monoterpenes exert activity of affecting emotions by directly acting on the olfactory nerve and central nervous system [38]. However, scarce detailed investigation was found in this area [39].

Thus, the aim of our investigation was to measure the effect of LEO on cognitive impairment mice with neurodegenerative disease by inhalation. The research questions and associated hypotheses to be tested included that an underlying mechanism to restore learning and memory in a degenerated brain would inhibit AChE to improve the neuroplasticity and upgrade the function of the protecting neurons. AD-related cognitive dysfunction is usually characterized by the loss of neurons and an elevated AChE level [40]. To establish the therapeutic potential of targeting AChE and synaptic plasticity, we used APP/PS1 transgenic mice and lemon essential oil. In this work, we examined the efficacy of the LEO in an animal model of AD, characterized by dysfunction of neurons and detected by memory decline after 4 months [41] and severe plaques formation after 6 months [42, 43]. The application of LEO ameliorates Aβ-induced impairment in synaptic plasticity and memory formation. Using in vivo approaches, we provide evidence that this effect is linked to the capacity of LEO to reduce AChE and contributes dramatically to its effects upon memory and synaptic plasticity. Until now, this is the first confirmation that essential oil effects, including synaptic plasticity and the synapse number, occur in the hippocampus of APP/PS1 transgenic mice, a model of cognitive dysfunction.

## RESULTS

### LEO decreases neuronal loss in APP/PS1 mice

**Figure 1:**
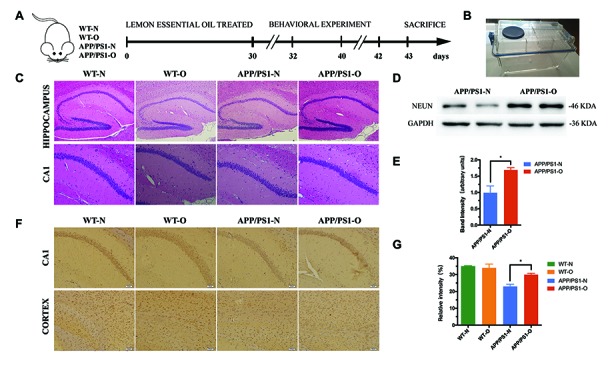
**LEO decreases neuronal loss in APP/PS1 mice.** (**A**) Experimental design. We treated mice with daily inhalation over a period of 30 days. After treatment, MWM was performed for 6 days, followed by Novel object recognition test for 3 days. All mice were then euthanized and pathological evaluations were performed. (**B**) Inhalation of LEO was performed using an individual ventilated cage (IVC) in the experiment. (**C**) H&E staining of the hippocampal sections from aged WT and APP/PS1 mice. (**D**) shows Western blotting data of NeuN protein expression levels in the hippocampus. (**E**) Bar graph represents NeuN expression levels in the hippocampus. (**F**) The neuronal mark protein NeuN was used to assess neuronal density in the CA1 region and cortex in WT and APP/PS1 mice. (**G**) Bar graph displays the number of NeuN-positive cells in cortex. Data are expressed as mean ± SEM. *P < 0.05, **P < 0.01, ***P < 0.001; one-way ANOVA with Tukey’s multiple comparisons test. Scale bar = 50 μm. LEO: lemon essential oil.

First, to study the effect of LEO on cognitive impairment, 8-month-old APP/PS1 mice and WT mice were housed in individual ventilated cages (IVCs) (Figure 1A and 1B) inhaling LEO for one hour for consecutive 30 days. The common neuronal marker protein NeuN was used to estimate neuronal density in the brain [44]. We examined NeuN expression in the hippocampus, the results revealed that levels of NeuN were significantly higher (*P* < 0.05) in LEO-treated APP/PS1 mice when compared with untreated APP/PS1 mice (Figure 1D and 1E). We next tested the effect of LEO upon apoptotic cell death in the hippocampal CA1 region and cortex using NeuN staining (Figure 1F), and quantification of cortical results is provided in Figure 1G. This result indicates that LEO play an important role in protecting the number of survival neurons in APP/PS1 mice.

Consistently, H&E staining results showed that neurons in the hippocampus region of WT mice were orderly arranged (Figure 1C) and had normal morphology. By contrast, many swollen neurons with a loose structure were observed in untreated APP/PS1 mice, while fewer pathological changes were observed in LEO-treated mice. The results indicated that LEO plays an important role in maintaining neuronal morphology. Interestingly, we also found that a reduced number of neurons in the olfactory bulb (OB) and hippocampus in LEO untreated APP/PS1 mice (Supplementary Figure 1A). The results displayed that the OB region neurons of WT mice had a normal structure, clear nuclei, and distinct nucleoli (Supplementary Figure 1A). However, many neurons with a loose structure can be found in untreated APP/PS1 mice, while fewer pathological changes were observed in LEO-treated APP/PS1 mice. Altogether, these results showed that LEO exerts an neuroprotective effect.

### LEO improves learning and memory ability after neuronal loss

**Figure 2:**
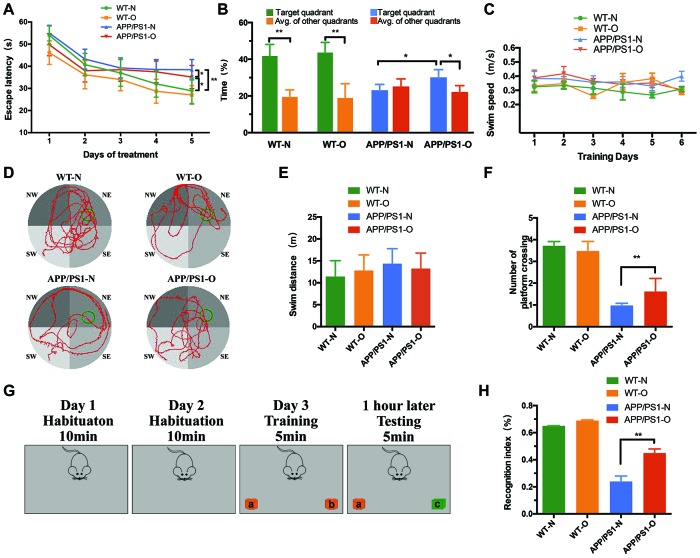
**LEO improves learning and memory ability in APP/PS1 mice after neurodegeneration. Escape latency and escape velocity in the MWM test were recorded and statistically analyzed.** (**A**) represents latency during the acquisition phase. Two-way repeated measures ANOVA revealed that APP/PS1 mice learned significantly slower than WT mice (*F* = 2.93, *P* = 0.0084). In APP/PS1 mice, the data revealed that the difference between LEO-treated mice and untreated APP/PS1 mice was significant (*F* = 2.96, *P* = 0.042). (**B**) represents percent time spent by mice in target quadrant and average of other quadrant and average of other quadrants during the probe trial. (**C**) represents swim speed during the acquisition phase. (**D**) represents video tracks of probe trial. (**E**) represents swim distance during the acquisition phase. (**F**) represents the number of crossing over the original platform location. (**G**) Schematic diagram of the Novel object recognition. (**H**) Recognition indices in mice were analyzed by one-way ANOVA (*P < 0.01*). Values are presented as means ± SD. *P < 0.05, **P < 0.01, ***P < 0.001. LEO: Lemon essential oil.

To investigate the effect of LEO on learning behavior after neuronal loss had already occurred, MWM and Novel object recognition test were performed. We found that, despite a comparable extent of neuronal loss (Figure 1), LEO-treated APP/PS1 mice demonstrated significantly increased associative and spatial learning compared with the nontreated APP/PS1 mice (Figure 2A). Untreated WT mice showed a standard learning curve in the MWM, as evidenced by a significant decrease in latency on day 5 (28.91 s) compared with that on day 1 (54 s) (Figure 2A). However, APP/PS1 mice demonstrated an impaired learning curve, as evidenced by no obvious difference in the latency between day 1 (55.01 s) and day 5 (38.46 s) (Figure 2A). Analysis showed a significant (*P* < 0.01) difference in the latency to reach the platform on day 5 between APP/PS1 and WT mice, suggesting an impaired learning ability of APP/PS1 mice. The motor performance appeared normal, as indicated by a nonsignificant difference in the mouse swim speed across the acquisition days (Figure 2C). The swim speed and swim distance of WT mice were not significantly different from those of APP/PS1 mice (Figure 2C and 2E). In the probe test, the APP/PS1 mice displayed a diminished MWM performance after final training, as measured by the number of crossing over the platform area (Figure 2F). APP/PS1 mice had a significantly higher proportion of figure “8” (Figure 2D) and large circling movements than LEO-treated mice. The average proximity from the platform was significantly (Figure 2D) higher in the APP/PS1 group than in the WT group, indicating that the untreated APP/PS1 mice retained a less accurate spatial memory (Figure 2B and 2D).

In order to further confirm the effects of LEO on short memory, we performed the Novel object recognition test. Figure 2G showed the protocol of the training day presenting two identical objects in the box. Then the testing day presenting one familiar object and a novel object. The results illustrated that there was no significant difference in location preference among four groups on training days. In the test phase, untreated APP/PS1 mice showed significantly lower recognition index compared with LEO-treated APP/PS1 mice (*P* < 0.01, Figure 2H). These results suggest that LEO can improve learning ability in AD mice with severe neurodegeneration.

### LEO suppressed accumulation of amyloid protein in APP/PS1 mice

**Figure 3:**
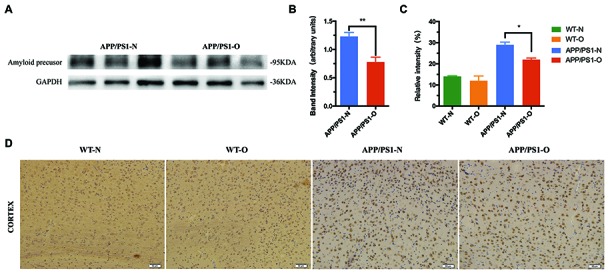
**LEO suppressed accumulation of amyloid protein in the brain.** (**A**, **B**) Expression of amyloid precursor of APP/PS1 mice. (**C**) Percentage of positively stained area in the cortex in four groups. (**D**) Representative photomicrographs of amyloid precursor immunoreactivity in the cortex was compared by one-way ANOVA (*P < 0.05*). Scale bar = 50 μm. Values are presented as means ± SEM. *P < 0.05, **P < 0.01. LEO: Lemon essential oil.

β - amyloid deposition is considered to be one of the characteristic pathological changes in AD. To test whether LEO can exert anti-amyloid effects, we treated APP/PS1 mice starting at 8 months of age with daily LEO inhalation over a period of 30 days. At the end of treatment, we analyzed the expression and distribution of amyloid precursor by using antibodies against aggregation-prone amyloidogenic proteins. The Western blotting results showed that LEO-treated APP/PS1 mice demonstrated a strong reduction (*P* < 0.01) in the components of amyloid deposits compared with untreated APP/PS1 mice (Figure 3A and 3B).

Then, we stained tissue sections with amyloid precursor in WT mice and APP/PS1 mice. We evaluated the degree of amyloid deposit of the cortex, which demonstrated that there are less difference between WT-N and WT-O group (Figure 3C and 3D). The IHC results matched the Western blotting results of APP/PS1 mice with large amount of amyloid deposit in the brain. Compared to WT mice, we found that APP/PS1 mice contain twofold higher levels of the Aβ (Figure 3D). In APP/PS1 mice, we observed that the cortical area covered by amyloid-β plaques was decreased (26%, *P* < 0.05) in LEO-treated APP/PS1 mice compared with untreated APP/PS1 mice (Figure 3D). This indicates LEO downregulate β - amyloid deposition in APP/PS1 mice. Representative images of the amyloid immunostaining specimens are shown for the four groups (Figure 3D).

### LEO increases ACh levels and downregulates AChE activity in the hippocampus

**Figure 4:**
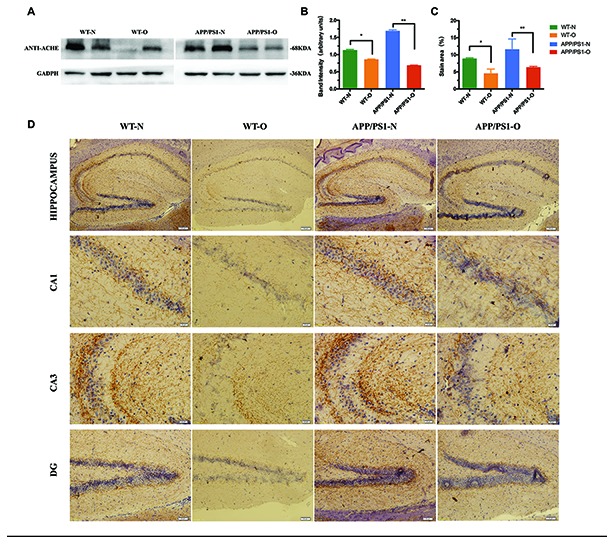
**Effects of LEO on acetylcholinesterase content in APP/PS1 mice and WT mice. Hippocampus of each group was extracted by extracting buffer and then estimated by Western blotting.** (**A**) Representative Western blotting of acetylcholinesterase. (**B**) Densitometric analyses of the immunoreactivity to the antibody shown in A. (**C**) Quantitative analysis for the relative intensity of AChE in hippocampus. (**D**) Immunostaining with anti-AChE antibody in each group. Scale bar, hippocampus = 100 μm, CA1, CA3 = 20 μm, DG = 50 μm respectively. Data are expressed as the means ± SD. *P < 0.05, **P < 0.01, ***P < 0.001, significantly different from non-treated mice group. LEO: lemon essential oil.

Cognitive impairments in neurodegenerative diseases have been related to reductions in ACh and increased levels of AChE in the hippocampus [45, 46]. Based on our finding of the MWM test and Novel object recognition test, we postulated that LEO improved learning and memory by reducing AChE activity and prolonging the action time of ACh. To test this hypothesis, we examined the AChE expression level in the hippocampus (Figure 4A) using Western blotting after 30 days of LEO treatment. We found that the AChE level of APP/PS1 mice was obviously elevated (31%) compared with that in WT mice (Figure 4B), indicating that APP/PS1 mice showed cholinergic system dysfunction. After LEO treatment, the AChE content of the hippocampus in APP/PS1 mice was significantly decreased. The expression of AChE was significantly increased (43%, *P* < 0.01) in untreated APP/PS1 mice compared with that in LEO-treated mice. The same result was observed in the WT group (*P* < 0.05) (Figure 4B).

Next, we examined the expression of AChE in the hippocampus by immunohistochemistry (IHC) using AChE antibody in WT and APP/PS1 mice (Figure 4D). The density of the AChE fibers in the hippocampal formation was decreased in the APP/PS1 mice compared with that in WT mice. IHC of the hippocampus CA3 and dentate gyrus (DG) was performed, and the results showed that AChE staining in APP/PS1 mice was increased compared with that in WT mice. We also found that LEO leads to a significant decrease in the number of AChE-positive cells in the CA1 compared with that in the untreated group, and quantitative analysis suggested that the contents of AChE decreased to 55 % (*P* < 0.01) in the untreated mice (Figure 4C). These data suggested that LEO treatment decreased the degeneration of the hippocampal cholinergic system by reducing AChE levels.

### The synapse number is significantly decreased in APP/PS1 mice and is repaired by LEO treatment

**Figure 5:**
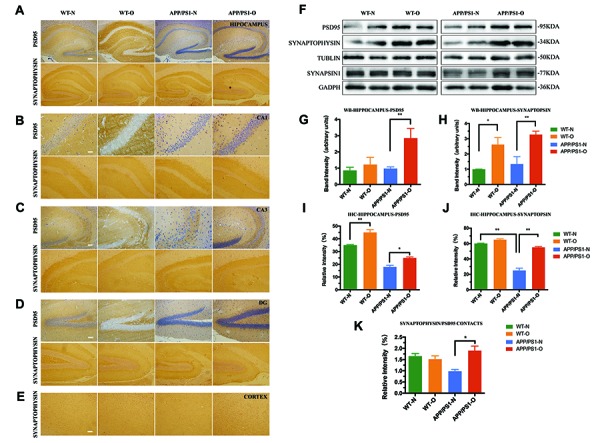
**Synaptic density is significantly impaired in APP/PS1 mice and repaired after LEO treatment.** IHC analysis for the expression and localization of both presynaptic marker, synaptophysin and postsynaptic marker, PSD95 in hippocampus (**A**), hippocampal CA1 region (**B**), hippocampal CA3 region (**C**) and hippocampal DG region (**D**), cortex region (**E**). (**F**) Western blot confirmation for PSD95, synapsin-1 and synaptophysin expression in hippocampus from APP/PS1 mice and WT mice. (**G**–**H**) Quantitative analysis of Western blots analysis for hippocampus. (**I**, **J**) Quantitative analysis for the relative intensity of synaptophysin, PSD-95, and SYN/PSD95 contacts which represents synaptic density (**K**) compared with WT mice. Scale bar, **A**–**D** = 100 μm, B = 20 μm, **C**–**E** = 50 μm. Values are means ± SEM. *P < 0.05, **P < 0.01, ***P < 0.001. LEO: lemon essential oil.

IHC staining for the presynaptic markers (IHC) staining for the presynaptic markers, synapsin-1 and synaptophysin, and postsynaptic marker, PSD95 was performed in both the hippocampal region and cerebral cortex (Figure 5A to Figure 5E) to visualize presynaptic and postsynaptic expression. Additionally, synapsin-1, which is associated with the regulation of neurotransmitter release from presynaptic neuron terminals [47, 48], is measured in the experiment (Figure 5F). APP/PS1 mice with no LEO treatment showed decreased synaptophysin immunoreactivity in the hippocampus compared with that in WT mice (*P* < 0.01) (Figure 5A and Figure 5J). Semiquantitative analysis of the IHC results demonstrated that untreated APP/PS1 mice exhibited a 39% increase in PSD95 (Figure 4I) and a 54% increase in synaptophysin (Figure 5J) in the hippocampus compared with those in untreated mice. Western blot analysis displayed a dramatic decrease in both the PSD95 (Figure 5G) and synaptophysin (Figure 5H) protein expression levels in the hippocampus of APP/PS1 mice compared with those in WT mice. The data also showed a significant increase in the PSD95 (65%, *P* < 0.01), synaptophysin (56%, *P* < 0.01) and synapsin-1 protein levels in the hippocampus of LEO-treated APP/PS1 mice compared with those in the untreated APP/PS1 mice (Figure 5F to Figure 5J).

Next, we examined whether the treatment with LEO could improve the impaired synaptic density in the hippocampus. IHC analysis showed that LEO treatment improved the expression of PSD95 and synaptophysin in APP/PS1 mice (Figure 5A to Figure 5E). The pyramidal neurons in the CA3 of the hippocampus showed a strong positive signal of PSD95 in the cytoplasm while CA1 (Figure 5B) and DG (Figure 5D) regions showed a strong positive signal of synaptophysin in the nucleus. The intensity of synaptophysin/PSD95 contacts PSD95 (Figure 5K) was observed as an index of reactive synaptic density, showing an 18% decrease in the hippocampal region of APP/PS1 mice compared with that in the WT group. The intensity of synaptophysin/PSD95 contacts displayed a 37% increase in the hippocampal region in LEO-treated APP/PS1 mice compared with that in untreated APP/PS1 mice. However, no significant difference was found between LEO-treated and untreated WT mice (*P* > 0.05). Together, these results demonstrated that the synapse number and synaptic density can be improved by LEO inhalation. Overall, these results indicated that LEO prompts to the restoration of memories by re-establishing the synaptic network.

### Neurotrophic signaling is significantly increased in LEO-treated APP/PS1 mice

**Figure 6:**
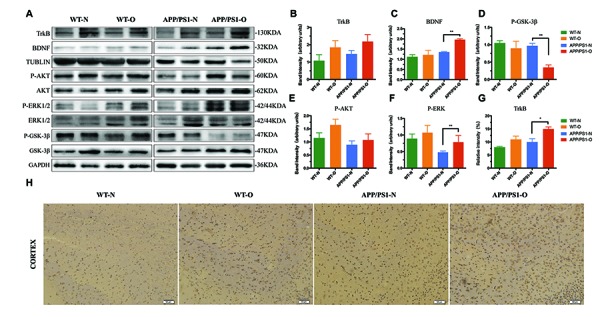
**Neurotrophic signaling is significantly increased after LEO treatment in APP/PS1 mice and WT mice.** (**A**) Hippocampus lysates were obtained after behavioral experiment and probed with antibodies detecting TrkB, BDNF, total GSK-3β, p-GSK-3β, total AKT, p-AKT, total ERK, p-ERK, total GSK-3β and p-GSK-3β levels in APP/PS1 and WT mice. Further quantitative analysis was conducted, with percentage changes versus WT mice shown in (**B**–**F**). (**G**) Immunostaining analysis result of **H**. (**H**) Immunostaining with TrkB antibody in each group. Values are means ± SEM of determinations from each group. *P < 0.05, **P < 0.01, ***P < 0.001. LEO: lemon essential oil.

Western blot results for synaptogenesis displayed that the expression of synapsin-1, synaptophysin and PSD95 in the brain was also robustly recovered by LEO in APP/PS1 mice (Figure 5). We next explored the mechanisms underlying the cognitive dysfunction improved by LEO via detecting the levels and activities of GSK-3β, which is considered a major regulator of cholinergic signaling. As presented in Figure 6A, LEO dramatically decreased ser-9-phophorylated GSK-3β expression in APP/PS1 mice compared with that in the untreated APP/PS1 group (63%, *P* < 0.01) (Figure 6D). The expressions level of total GSK-3β was constant in the control and experimental groups. We found that LEO did not induce obvious change in the protein levels of the ser-9-phophorylated GSK-3β in the WT group (*P* > 0.05).

Additionally, considering that the presynaptic protein synaptophysin is a downstream target of the BDNF pathway, we sought to explain the regulating mechanism of LEO in hippocampal synaptogenesis. To accomplish this, we next tested TrkB-BDNF and rapid AKT-ERK kinase neurotrophic signaling in the brain of APP/PS1 mice and WT mice. Western blot data demonstrated that the phosphorylation levels of AKT and ERK in the hippocampus of LEO-treated APP/PS1 mice was increased by 20% (*P* > 0.05) and 63% (*P* < 0.01) compared with those in untreated APP/PS1 mice (Figure 6E and 6F). Likewise, studies in LEO-treated WT mice revealed a 43% increase in pAKT and 20% increase in pERK levels in the hippocampus, compared with those in untreated WT mice (Figure 6E and 6F). Results showed that the TrkB in LEO-treated APP/PS1 mice was rescued in the hippocampus and cortex by LEO (Figure 6A and 6H), while the BDNF levels were increased significantly (Figure 6C, *P* < 0.01), indicating a restoration of these signaling pathways. This appears to be a true “restore" and not just enhancement regulation by LEO in the brain because LEO could to restore the BDNF levels and phosphorylation of AKT and ERK in APP/PS1 mice but had no obvious effect in WT mice (Figure 6A), and APP/PS1 mouse brain may be more sensitive to LEO. The results suggest that the beneficial effect of LEO may be associated with its enhancement of prominent plasticity.

## DISCUSSION

**Figure 7:**
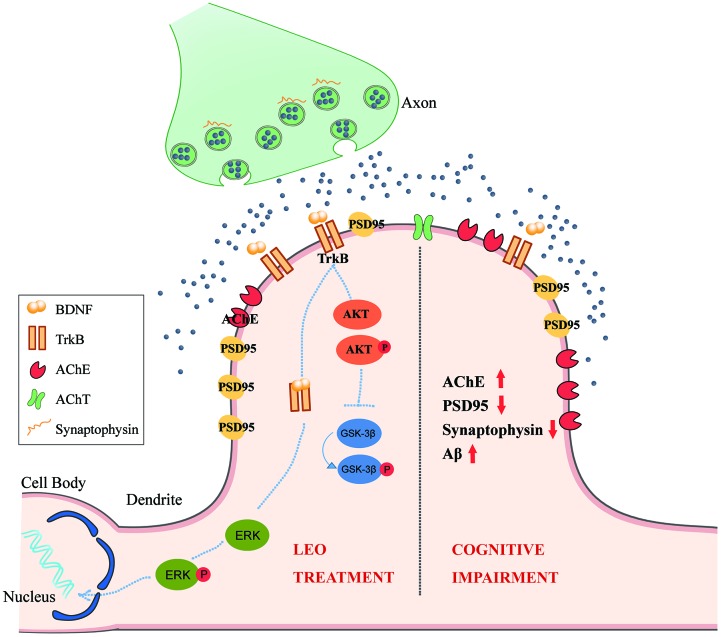
**The hypothesis model of the signaling pathways by which LEO regulates neuroplasticity in cognitive deficits.** In the pathological state of AD, AChE marker protein is up-regulated in the CA1 pyramidal neuron of APP/PS1 mice. PSD95, Synaptophysin, Synaptophysin /PSD95 contacts is also decreased in APP/PS1 mice. After LEO treatment, both presynaptic marker, synaptophysin and postsynaptic marker, PSD95 in hippocampus were up-regulated. LEO was demonstrated to be important for activation of BDNF, AKT and GSK in the hippocampus of AD mice. LEO may increase hippocampal neuroplasticity via acting upon three signaling pathways: (1) LEO may up-regulate BDNF protein level in the hippocampus, BDNF binding to its high-affinity TrkB, associated with neuroplasticity, including persistence of cell survival, stimulation of synaptic plasticity; (2) LEO may activate AKT, which is the downstream of TrkB receptor stimulation and related to enhancement of synaptic plasticity. (3) LEO could improve cognitive impairment by decreasing the AChE contents. GSK-3β can regulate the AChE levels in pathological conditions. AD, Alzheimer’s disease; ACh, acetylcholine; AChE, choline acetyltransferase; TrkB, receptor tyrosine kinase; AKT, protein kinase B; ERK, extracellular signal-regulated kinase; LEO, lemon essential oil.

In our work, we investigated the effect of LEO in the established APP/PS1 mouse model of AD and WT mice. The APP/PS1 mouse model is characterized by dysfunction of neurons, and visible plaque deposition is detected after 6 months [49]. The most important findings of the present research were that LEO treatment partly restored hippocampal synaptic plasticity in LEO treated APP/PS1 mice. In our study, we found that 4 weeks of LEO treatment partly reversed the spatial reference memory impairment of 8-month-old APP/PS1 mice. An increased the expression of BDNF, TrkB and phosphorylated AKT and ERK, including the levels of marker proteins for synaptic integrity and plasticity that could induce the restoration of spatial memories. LEO-treated APP/PS1 mice also displayed a significant decrease in AChE in the hippocampus, which would increase ACh levels. These results are summarized in Figure 7. In line with this observation, spatial reference memory was partially restored in the LEO-treated APP/PS1 group. Many preclinical studies have attempted to restore synaptic plasticity and memory function in mouse models of amyloid pathology [41]. This is the first confirmation that LEO effects, including synaptic plasticity and the synapse number, occur in the hippocampus of APP/PS1 mice, a model of cognitive dysfunction. Importantly, LEO has few side effects and can penetrate well into the BBB [21, 50]. Some studies have shown that the permeability of essential oil can enhance the signal transmission [51], and some components of essential oil can increase the permeability of water, which can be widely used in industrial production [52]. Overall, the findings in this investigation suggest an important potential role for LEO as a therapeutic agent to restore memory function in AD.

Neurological degenerative diseases such as AD caused by aging can lead to impairment of neurological function, especially spatial memory [53]. The MWM test is a classical experiment to measure animal space ability and can effectively demonstrate the ability of animal spatial and memory learning [54, 55]. In the study, the MWM was used to directly explain spatial memory formation and estimate spatial learning ability. Our results revealed that LEO-treated APP/PS1 mice showed better spatial memory ability. The influence of LEO on the motor function of mice was also studied using the MWM test. In our study, the swimming speed of APP/PS1 mice was similar to that of untreated WT mice. Our study showed that a low dose LEO (1 ml/cage) would not influence the movement of normal mice and APP/PS1 mice, ensuring the safe use of LEO. Spatial and contextual memory appears to depend on the dorsal hippocampus [56, 57]. It has been reported that CA1 in the hippocampus is sensitive to the neurotoxicity of Aβ. Under the action of CA1, the hippocampus is prone to degenerative lesions [58]. In line with this, our current anatomical analyses using immunohistochemistry analysis provide new evidence that APP/PS1 mice have defects in the hippocampal region, specifically the CA1 region. H&E staining demonstrated more neurons in the LEO-treated group than in the untreated group, indicating the protective effect of LEO on neurons. Additionally, LEO has no significant effect on the morphology of nerve cells in brain tissue, providing a theoretical basis for the safe use of LEO.

One of the most important findings of the our study was that LEO treatment restored learning and memory in the APP/PS1 mice by decreasing the expression of AChE. We investigated the effect of LEO in the established APP/PS1 mouse model of AD and WT mice by analyzing the expression of AChE. Previous reports have demonstrated increases in AChE levels in AD before neuronal degeneration [59]. Cholinergic transmission is terminated by ACh and can be hydrolyzed by AChE in the synaptic cleft [60]. The change in the AChE contents is the primary index to report the biochemical changes of cholinergic energy in AD indirectly [59]. In our study, LEO significantly decreased AchE protein levels in the hippocampus compared with that in the untreated group. Similarly, the levels of AChE in the hippocampus of WT mice were also dramatically decreased after LEO treatment, indicating that LEO could improve memory by decreasing the content of AChE in the hippocampus. Under some pathological conditions, GSK-3β activity is upregulated, exerting its adverse influence on cholinergic signaling increasingly and, at the same time, inducing phosphorylation events critical to the development of proteinopathies [61]. Thus, we explored the relationship underlying cognitive improvement by LEO via detecting the levels and activities of GSK-3β, which is considered the major regulator of cholinergic signaling. Although we did not test the levels of LEO components in the blood of mice, our studies revealed that inhalation of the LEO apparently affected the AChE content in the hippocampus, suggesting that several active components affected the GSK-3β signaling pathways in the CNS.

One of our main questions is whether synaptic plasticity is involved in the cognitive improvement in LEO-treated APP/PS1 mice. Cytotoxicity caused by Aβ can destroy the connections between synapses and directly affect normal cognitive function [7, 62]. Similarly, our results showed a significant decrease in the synaptic density in the brain of APP/PS1 mice compared with that in LEO-treated mice. Our investigation also revealed that treatment of APP/PS1 mice with LEO significantly improved the impairment in synaptic transmission efficiency partially through increasing the expression of PSD95, synaptophysin and synapsin-1 protein. Indeed, a previous study displayed that LEO can enhance synaptic plasticity in the rodent brain. This is the first time revealed that essential oil can enhance the synaptic plasticity and synapse number. We will examine the LTP (which plays a critical role in the formation of certain forms of memory) of APP/PS1 mice in future experiments. Collectively, our studies highlight the importance of LEO in maintaining the PSD95, synapsin-1 and synaptophysin levels in the brain.

Our discovery also provided insight into the potential signaling mechanisms that underlie the effects of LEO on memory and synaptic plasticity. Our studies revealed that AKT and ERK activation were obviously attenuated in the brain of APP/PS1 mice, indicating that LEO can mediate these rapid kinase signaling pathways. Recently, both AKT and ERK signaling has been explained to help regulate the LTP, memory and synaptic plasticity [63–65]. Thus, we propose that LEO mediates AKT and ERK activation in the brain, which may contribute dramatically to its effects on the LTP, synaptic plasticity and memory of APP/PS1 mice. Our findings demonstrated an obviously decrease in the TrkB and BDNF levels in the APP/PS1 brain and a significant increase in the TrkB and BDNF levels after LEO treatment in APP/PS1 mice. BDNF is a neurotrophin that plays a critical role in the modulation of the LTP, memory and synaptic transmission through the effects regulated by TrkB receptors that are coupled to activation of the ERK and AKT pathways [66–69]. Furthermore, BDNF knockout mice are characterized by impaired LTP, memory and synaptic plasticity [70], findings that are consistent with ours in APP/PS1 mice. Taken together, our discovery revealed the critical role of LEO in maintaining BDNF levels, as well as the activation of TrkB, AKT and ERK in the brain of APP/PS1 mice.

Collectively, our experiments showed that LEO can regulate synaptic plasticity and hippocampal-dependent memory function in APP/PS1 mice and WT mice. Although further experiments will be provided to uncover the detailed LTP experiments of LEO’s function on cognitive deficits in mice, our data clearly revealed that inhaled LEO suppresses the impairment of spatial learning and synaptic loss caused by the expression of synaptophysin. LEO could partially rescue the ACh levels in a GSK-3β dependent manner. LEO was also shown to be critical to maintain the BDNF levels, as well as activation of ERK and AKT in the brain of APP/PS1 mice. For the first time, our discoveries offer in vivo evidence that LEO provides protection against AD-related synaptic loss and memory impairment. Our finding also supports the importance of LEO as a “memory enhancer” in both the WT and APP/PS1 brain to mediate AChE, synaptic plasticity and cognitive function.

## MATERIALS AND METHODS

### Inhalation and animals

Eight-month-old male C57BL/6 and APP/PS1 mice were obtained from the Beijing Vital River Laboratory Animal Technology Co., Ltd. and were housed as five mice per cage under controlled temperature (22 ± 2°C) and humidity (50 ± 5%) on a normal 12-h light/ dark cycle with food and water ad libitum. All experimental procedures were conducted in according to the guidelines relevant to the Care and Use of Laboratory Animals published by the US National Institutes of Health. LEO was purchased from Shanghai Yuanye Bio-Technology Co., Ltd. Inhalation of LEO was conducted using an individual ventilated cage (IVC), which was made of acrylate board (33 cm × 20 cm × 18 cm) and could vaporize and distribute LEO evenly. APP/PS1 and WT mice were randomly assigned into one of four groups respectively: the WT water-treated group (WT-N group, n = 12), the WT LEO-treated group (WT-O group, n = 12), the APP/PS1 mice water-treated group (APP/PS1-N group, n = 12), and APP/PS1 mice LEO-treated group (APP/PS1-O group, n = 12). We treated mice starting at 8 months of age with water or LEO (1 mL/cage) inhalation in the IVC for 1 hour over a period of 30 days. After treatment, MWM was performed for 6 days (n = 12), followed by Novel object recognition for 3 days (n = 6). Thereafter, the mice were euthanized and pathological evaluations were performed in all groups.

### Morris water maze test

Mice were single-caged and brought into the testing room prior to the beginning of the experiment. The MWM test was conducted in a circular tank (diameter, 140 cm; height, 50 cm) (Shanghai Mobildatum Technology Co., Ltd.) in a dimly lit room. The water temperature was kept at 22-25°C to inhibit the mice from floating. A submerged escape platform (10 × 10 cm) was equipped 1.5 cm below the milky water surface in one of the quadrants. Spatial cues of different geometry were decorated by the pool sides to help the mice recognize the platform position. The mice were individually handled for 1 day before starting the acquisition training. The mice were trained over 5 consecutive days with four trials per day per mouse. The trial was completed as soon as the mouse found the platform or when 60 seconds had elapsed. If the mouse cannot find the submerged platform on a given trial, the mouse was guided to the submerged platform. The latency and path to the platform were tracked and recorded. The swim speed was measured to analyze the involvement of motor function as a confounding factor. On day 6, a single probe test was performed to measure the integrity and strength of spatial memory 24 hours after the last trial of the acquisition phase. The results in the probe trial were measured by analyzing the time spent by APP/PS1 and WT mice in the given quadrant and the average proximity to the escape annulus.

### Novel object recognition test

Novel object recognition test was a method for learning and memory test, based on the principle that mice have instinct to explore new objects. The experimental installation was made up of rectangular box and three objects (A, B and C), of which A was same to B, while the object of C was obviously diverse from the A and B. On the first and second day, the mouse (n = 6) being measured was habituated to the area for 10 min. On the third day, we placed the mice in the opaque box together with two objects (A and B) and allowed it to explore for 5 min, and then returned the mouse to the cage. After 1 hour, the B was replaced with C in the same position, and the mouse was placed back to the box and allowed to explore for 5 min. The zone and objects should be washed with ethanol. The recognition index was analyzed by dividing the amount of time spent exploring the novel object by the total time exploring both objects in the test.

### Hippocampus lysates preparation and western blotting

Hippocampal tissue was incubated on ice for half an hour in ice-cold RIPA buffer (Beyotime), containing phosphatase inhibitors. Thereafter, centrifugation was performed at 12,000 g for 15 min to obtain total protein fractions in the supernatant. The protein concentrations were measured using the BCA Protein assay kit (Beyotime). The supernatants were stored at -80°C for Western blotting until ready for use. The primary antibodies used for Western blotting analysis included the following: Rabbit anti-PSD95 (Cell Signaling, D74D3, 1:1000), Rabbit anti-synapsin-1 (Cell Signaling, D12G5, 1:1000), Mouse anti-synaptophysin (Abcam, ab8049, 1:1000), Rabbit anti-Acetylcholinesterase (Abcam, ab183591,1:1000), Rabbit anti-BDNF (Wanlei, 1:500), anti-p-AKT (Wanlei; 1:500), anti-AKT (Wanlei; 1:500), anti-p-ERK/1/2 (Wanlei; 1:500), anti-ERK1/2 (Wanlei; 1:500), Mouse anti-GSK-3β (Bioss, bsm-33293M, 1:1000), and Mouse anti-GAPDH (ZSGB-BIO, 1:2000). Specific protein bands on the membranes were measured with a 1:5,000 dilution of the appropriate HRP-conjugated (ZSGB-BIO) secondary antibody and were visualized by enhanced chemiluminescence (Beyotime) and quantified by an image analyzer (ImageJ software).

### H&E staining

Each mouse was recognized according to the analysis of tail DNA by PCR to distinguish the APP/PS1 and WT mice. The mice (n = 3) were perfused via the tips of the heart with phosphate-buffered saline (PBS, pH 7.2) and 4% paraformaldehyde in PBS. The mouse brains were collected and maintained in 4% paraformaldehyde for 48 hours and processed for paraffin embedding. The brains were serially sectioned sagittally at 4 μm using a microtome. Parasagittal sections (4-μm thick) of the brain were stained with hematoxylin and were used for H&E staining.

### Immunohistochemistry and imaging

IHC was conducted using a commercial avidin-biotin complex kit (ZSGB-BIO), as shown in a previous study with slight modifications [71]. Deparaffinized sections were treated with 0.01 M citrate buffer (pH 6.0) in an antigen retrieval instrument for 1.5 hours (Thermo). After blocking with 5% BSA for 20 minutes at room temperature, the sections (n = 3) were incubated for 24 hours with primary antibody at 4 °C in a humid chamber. After three washes in PBS, the sections were further incubated with anti-mouse or anti-rabbit biotinylated secondary antibodies for 20 minutes at 37 °C and then were stained using the DAB kit (ZSGB-BIO) for 1 minute. Next, the sections were cover slipped with a neutral balsam mounting medium.

These primary antibodies were used: Rabbit anti-PSD95 (Cell Signaling, D74D3, 1:1000), mouse anti-synaptophysin (Abcam, ab8049, 1:1000), Rabbit anti-Acetylcholinesterase (Abcam, ab183591, 1:1000), Rabbit anti-Amyloid precursor (Abcam, ab32136, 1:800), mouse anti-TrkB (Wanlei, WL00839, 1:200). These sections were mounted on microscope slides and were imaged using Olympus FSX100 microscope. We analyzed 3-5 consecutive brain sections for staining markers from each mouse. The images were analyzed with ImageJ software. Assessment of the PSD95 and synaptophysin intensities to indicate the numbers of presynaptic puncta and postsynaptic puncta respectively was analyzed by Image software. The synaptic density in the hippocampal region was presented as percentage changes [72].

### Statistical analysis

The data were displayed as the means ± standard error of the mean (SEM) or ± standard deviation (SD) in the expression of various biochemical markers (by Western blot and immunochemistry). Statistical significance of differences between groups was analyzed using two-way analysis of variance (ANOVA) followed by Tukey’s multiple comparisons test to determine the individual and interactive effects of FUS on behavioral analysis. In the MWM, we averaged the escape latencies across the four trials for each mouse. These means were analyzed across five sessions. A two-way repeated measures ANOVA was used for main effect with sessions as the repeated measure and escape latency as the dependent variables. *P* < 0.05 was considered significant. Analysis was performed using GraphPad Prism software.

## Supplementary Material

Supplementary Figure 1
