# Supplementary material for: Lemon essential oil ameliorates age-associated cognitive dysfunction via modulating hippocampal synaptic density and inhibiting acetylcholinesterase
Source: Aging (Albany NY). 2020 May 11;12(9):8622–39. doi: 10.18632/aging.103179 (PMC7244039; doi:10.18632/aging.103179)
Supplement: Supplementary Figure 1 [file aging-12-103179-s001..pdf]

SUPPLEMENTARY FIGURE

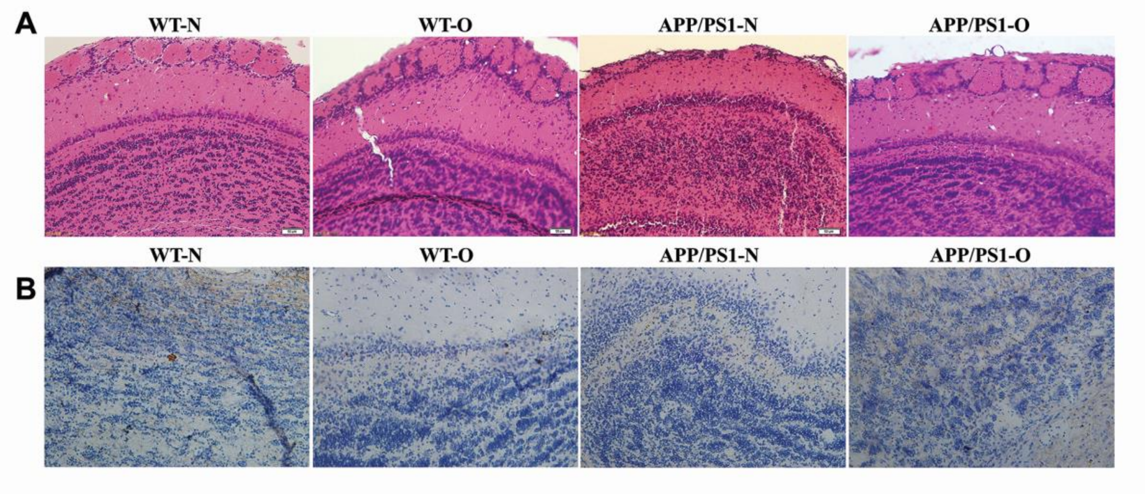

Supplementary Figure 1. LEO may play a role through the olfactory system. (**A**) H&E staining of OB in APP/PS1 and WT mice. (**B**) Immunostaining with anti-synaptophysin antibody in APP/PS1 and WT group. LEO: lemon essential oil.
